# The costs of traumatic brain injury due to motorcycle accidents in Hanoi, Vietnam

**DOI:** 10.1186/1478-7547-6-17

**Published:** 2008-08-22

**Authors:** Hanh TM Hoang, Tran L Pham, Thuy TN Vo, Phuong K Nguyen, Christopher M Doran, Peter S Hill

**Affiliations:** 1 Institute for Health Strategy and Policy, Ministry of Health, Vietnam; 2 University Training Center for Health Care Professionals, Ho Chi Minh City, Vietnam; 3 National Drug and Alcohol Research Centre, University of New South Wales, Australia; 4 School of Population Health, The University of Queensland, Australia

## Abstract

**Background:**

Road traffic accidents are the leading cause of fatal and non-fatal injuries in Vietnam. The purpose of this study is to estimate the costs, in the first year post-injury, of non-fatal traumatic brain injury (TBI) in motorcycle users not wearing helmets in Hanoi, Vietnam. The costs are calculated from the perspective of the injured patients and their families, and include quantification of direct, indirect and intangible costs, using years lost due to disability as a proxy.

**Methods:**

The study was a retrospective cross-sectional study. Data on treatment and rehabilitation costs, employment and support were obtained from patients and their families using a structured questionnaire and The European Quality of Life instrument (EQ6D).

**Results:**

Thirty-five patients and their families were interviewed. On average, patients with severe, moderate and minor TBI incurred direct costs at USD 2,365, USD 1,390 and USD 849, with time lost for normal activities averaging 54 weeks, 26 weeks and 17 weeks and years lived with disability (YLD) of 0.46, 0.25 and 0.15 year, respectively.

**Conclusion:**

All three component costs of TBI were high; the direct cost accounted for the largest proportion, with costs rising with the severity of TBI. The results suggest that the burden of TBI can be catastrophic for families because of high direct costs, significant time off work for patients and caregivers, and impact on health-related quality of life. Further research is warranted to explore the actual social and economic benefits of mandatory helmet use.

## Background

Each year an estimated 1.2 million people die and a further 20–50 million are injured worldwide from road traffic accidents: a major public health problem [1]. In Vietnam, road traffic injuries are now the leading cause of fatal and non-fatal injuries [2].

Motorcycle users in Vietnam are most vulnerable to road traffic injuries. Motorcycles account for approximately 95% of the total number of vehicles in Vietnam [1]. In 2001, there were an estimated 105 motorcycles per 1,000 population, increasing to 193 by 2005. Such an increase in motorcycle use has had significant effects on the burden of injury from road traffic injuries and the economic costs of treatment and the sequellae of injury. A community-based survey undertaken in 2001 in all eight regions of Vietnam showed that motorcycle users accounted for 51.3% of all non-fatal road traffic injuries, a rate of 734 per 100,000 population [2].

According to the World Health Organization, traumatic brain injury (TBI) is the main cause of fatal and non-fatal injury for motorcycle users [1]. In poor countries, economic losses caused by TBI due to expenditure for prolonged treatment, loss of productivity or income due to disability or death commonly tip households into a spiral of poverty [3]. No hospital-based or community epidemiological data on TBI in motorcycle users are available in Vietnam. However, it is likely that the burden caused by TBI to the country is significant, given the very low use of motorcycle helmets and the dominance of motorcycles as the main form of transport.

Mandatory motorcycle helmet use is regarded as the single most effective approach for the prevention of TBI among motorcycle users in both developed and developing countries [1]. Wearing a helmet reduces the incidence, severity and mortality rates of TBI in motorcycle accidents, ranging from 20% to 45% reduction of fatal and serious head injury [4]. In Vietnam, a mandatory helmet law was introduced for all roads on 15 December 2007, two years after this study was completed. Prior to this, it was mandatory to wear a helmet only on selected roads, mainly those designated as national roads, but the enforcement of that policy was poor. Nationwide, in 2001, only 7.4% of male and 4.1% of female regular motorcycle users reported using a helmet [5]. At the time of writing, compliance with mandatory helmet use appears high, though issues of helmet quality have been raised.

This study estimates the costs of non-fatal TBI in motorcycle users not wearing helmets in Hanoi, Vietnam, in their first year post-injury. The study examined costs from the perspective of the injured patients and their families. These included direct costs associated with treatment at hospital and at home; indirect costs associated with the loss of productivity; and intangible costs associated with the loss of quality of life. Although the social perspective is considered the most appropriate viewpoint to adopt in economic evaluations [6], this was not possible, due to the lack of available data and the currently limited role of health and social insurance in Vietnam.

## Methods

The study was undertaken at VietDuc Hospital, Hanoi, the major trauma centre in North Vietnam. Patients discharged between January 2005 and mid July 2005 with a history of TBI were enrolled in the study, based on the following inclusion criteria: aged 16 years and over; residential address in Hanoi; discharged at least 6 months before the commencement of the study; motorcycle driver or passenger not using a helmet when the accident happened; and no other serious injuries, complications or compounding diseases. Patients were further classified into three levels of TBI severity according to the Glasgow Coma Scale (GCS) at admission: severe (< 9), moderate (9–12) and minor (13–15).

### Cost analysis methods

Direct and indirect costs were quantified in economic terms. The direct cost method was used to estimate the costs associated with treatment, including household expenditure on all goods and services relating to the medical care of patients. The human capital method was used for the calculation of loss of productivity for the injured and their carers [7-10]. Although valuing the intangible costs of injury is difficult and often contentious [1], the health status index method was chosen for the assessment of the health-related quality of life, using years lived with a disability (YLD) as a proxy. The intangible costs, in this case, were *not *estimated in economic terms.

Structured questionnaires were used to obtain costs associated with the treatment of TBI, and productivity loss. For direct costs, respondents were asked to recall medical and non-medical costs at all health facilities and at home. Where interviews occurred less than one year following injury, projections of costs of home care to one year were estimated, based on current patterns.

Loss of productivity for patients and caregivers (indirect costs) were quantified in monetary terms using both individual actual income and per capita income for urban areas in Vietnam in 2004 (VND 815,000/month, equivalent to USD 51.5 in 2004). As with direct costs, for patients less than one year post-injury, projections of time off work were based on averages for each severity level. In the severe category, patients who had lost more than the mean number of weeks work at interview were assumed to be incapable of resuming work for the remainder of the year. The opportunity costs for loss of normal activity in students, the elderly or un-paid home-makers were estimated using the national per capita income in 2004 (VND 484,000, equivalent to USD 30.5).

The European Quality of Life instrument (EQ6D) instrument was translated, back translated and trialed, then used in patient interviews to measure changes in quality of life for discharged patients. This instrument uses six dimensions of health: mobility, pain/discomfort, self-care, anxiety/depression, usual activities and cognition [11]. Health status for each patient was represented by a single index with 6 digits. This was converted into a predicted disability weight (DW) under the "Disability Adjusted Life Years (DALY) form" using the Dutch Disability regression model ranging from "zero" for good health to "one" for death [11]. The YLD caused by TBI in one year was then calculated using the basic formula applied by The Global Burden of Disease and Injury: YLD = I × DW × L where I is the number of accident cases in the reference period, L is the average duration of disability [12]. In this case, the YLD of one patient with TBI was: 1 (case) × the predicted DW × 1 (year) with an assumption that the health state assessed at interview was representative of the patient's health state for one year post-injury.

Each cost component was calculated by three levels of TBI severity. The ANOVA test was used to compare the variance of the three level averages.

## Results

### Demographic characteristics of study population

Discharge records from VietDuc hospital showed 61 patients met the inclusion criteria. Initial telephone contacts with these 61 patients and their families showed that five patients were deceased, ten were not contactable or had relocated, and three were wearing helmets at the time of the accident. In two cases, motorcyclists were injured as a result of inter-personal violence, rather than motorcycle related incidents. Six patients refused to participate, and the remaining 35 patients were recruited to the study. Four of these had exceptional insurance or other third party financial support. As a result, total treatment costs and lengths of stay were extremely high in comparison with the remaining cases in the same level of severity, and these cases were considered as outliers for the purposes of this study.

Seventy one percent (22/31) of the study population was male. The mean age for the group was 33.2 years, with almost half (45.2%) between 20 to 29 years. Students accounted for 22.6%, followed by manual labourers in the industry/processing/handicraft sector (16.1%). Three-quarters (74%) were motorcycle drivers at the time of the accident. The GCS based severity of injury was evenly distributed: severe (10), moderate (11) and minor (10).

### Direct costs

Severity of injury correlated directly with length of stay at health facility and length of medication-use at home respectively: severe (3.2 week and 35.9 weeks); moderate (2 weeks and 17.5 weeks); and minor (2 weeks and 15.3 weeks). Similarly, direct costs, both in hospital and at home, increased with the severity of TBI (Table 1).

**Table 1:** One-year costs associated with treatment by level of traumatic brain injury *(Unit: USD, 1USD = 15,850 Vietnam dong)*

	**Level of severity by GCS**								
	
	**Severe**			**Moderate**			**Minor**		
	
	Mean	SE	Median	Mean	SE	Median	Mean	SE	Median
At all health facilities (*)	1571.3	285.9	1313.9	1060.3	156.5	1205	708.3	104.7	789.3
At home (*)	793.4	135.1	737.3	329.9	86.8	227.4	140.7	43.6	94.6

Total (*)	**2364.7**	**336.6**	**2201.3**	**1390.1**	**132.4**	**1457.4**	**849.0**	**110.0**	**861.8**

(*) Anova: p < 0.05

Costs at home included medication (including tonics and "therapeutic" foods) and rehabilitation in the form of physical therapy to improve health status. The low values for rehabilitation reflect the limited resources available to families, and their limited accessibility. Costs for ongoing home visits by therapists are not financially sustainable in this population. Although home treatment costs rose with severity, they remained substantially less than hospital costs at all levels. The use of rehabilitation services at home or though out-patient attendance was minimal: only four cases reported post-discharge rehabilitation services, accounting for a minor component of overall home costs.

### Indirect costs

The post-injury period was marked by a diminished ability to work or to conduct normal activities. Sixty percent of patients suffering severe TBI could not resume work or implement their usual daily activities again after 6 months. In the moderate group, twenty percent had persisting disability at this point, though all minor injury patients had returned to normal functionality. Where patients returned to work, it was frequently at lower levels of productivity with commensurate reductions in salary levels. Twenty percent lost their pre-injury role of family primary income earner.

Eighty-percent of discharged patients in the sample needed support from a caregiver at home after the accident. Where possible, households were strategic in minimizing the loss of household income by selecting caregivers with the lowest earning capacity in the family. In 35.5% of households, care-givers were non-working family members or the very old (home-makers, retired, unemployed or students). For 45.2% of caregivers, their income pre-injury was less than the national per capital income (USD 30.5 in 2004), and in 64% of cases, the selected caregiver had an income less than the capita income for urban areas (USD 53 in 2004). The withdrawal of a child from school to provide care for an injured adult or to work in order to compensate for lost income, represents a substantial opportunity cost, not reflected in the calculations of income foregone. Despite efforts to minimize income lost, opportunity costs for households from providing care were significant, and caregivers were not always available – accounting for the substantial difference between time loss for the injured and their caregivers (Table 2).

**Table 2:** Time off work or normal activity by level of severity of traumatic brain injury (Unit: weeks)

**Time off work or normal activity**	**Level of severity by GCS**								
	
	**Severe**			**Moderate**			**Minor**		
	
	Mean	SE	Median	Mean	SE	Median	Mean	SE	Median
By patient	38.4	4.8	40.3	18.9	5.1	13	11.5	2.2	13
BY caregiver	15.5	3.1	11.2	7.1	1.4	7.4	5.5	1.4	4.3

Total (*)	**54.0**	**6.9**	**59.5**	**26.0**	**6.2**	**20**	**17.1**	**3.0**	**21**

(*) Anova: p < 0.05

Estimates of loss of productivity using the individual's actual income produced average indirect costs that were much higher than the estimates based on per capital income for urban areas (Table 3). The advantage of using per capita income to estimate lost productivity, instead of the actual (known) income, is that it eliminates the variation of income evident in small samples. For both estimates, the loss of productivity rose with severity, though using per capita income the estimated losses were more conservative.

**Table 3:** Loss of productivity estimates using actual income and per capita income for urban area *(Unit: USD, 1USD = 15,850 Vietnam dong, per capita income for urban area was VND 840,000 per month)*

**Indirect costs for one year**	**Level of severity by GCS**								
	
	**Severe**			**Moderate**			**Minor**		
	
	Mean	SE	Median	Mean	SE	Median	Mean	SE	Median
***Actual income based indirect costs***									

By patient	1108.6	324.2	833.4	303.0	85.1	164.0	461.3	171.9	200.3
By caregiver	303.8	129.3	132.9	102.0	31.4	60.9	77.4	27.1	40.9

Total (*)	**1412.4**	**366.5**	**1045.3**	**405.0**	**106.2**	**239.0**	**538.5**	**194.0**	**263.5**

***Per capita urban income based indirect costs***									

By patient	455.8	56.5	478	223.7	60.6	154.2	136.4	25.7	154.2
By caregiver	183.5	36.6	132	84.0	17.0	87.8	65.4	17.1	50.9

Total(*)	639.4	82.2	702.5	307.8	73.1	242.0	201.8	35.7	245.4

(*) Anova: p < 0.05

^1^General Statistic Office: The Vietnam Living Standard Survey in 2004 showed that per capita income for urban area in 2004 was VND 840,000 per month; national per capita income was VND 484,000 per month.

### Intangible costs

**Figure 1:**
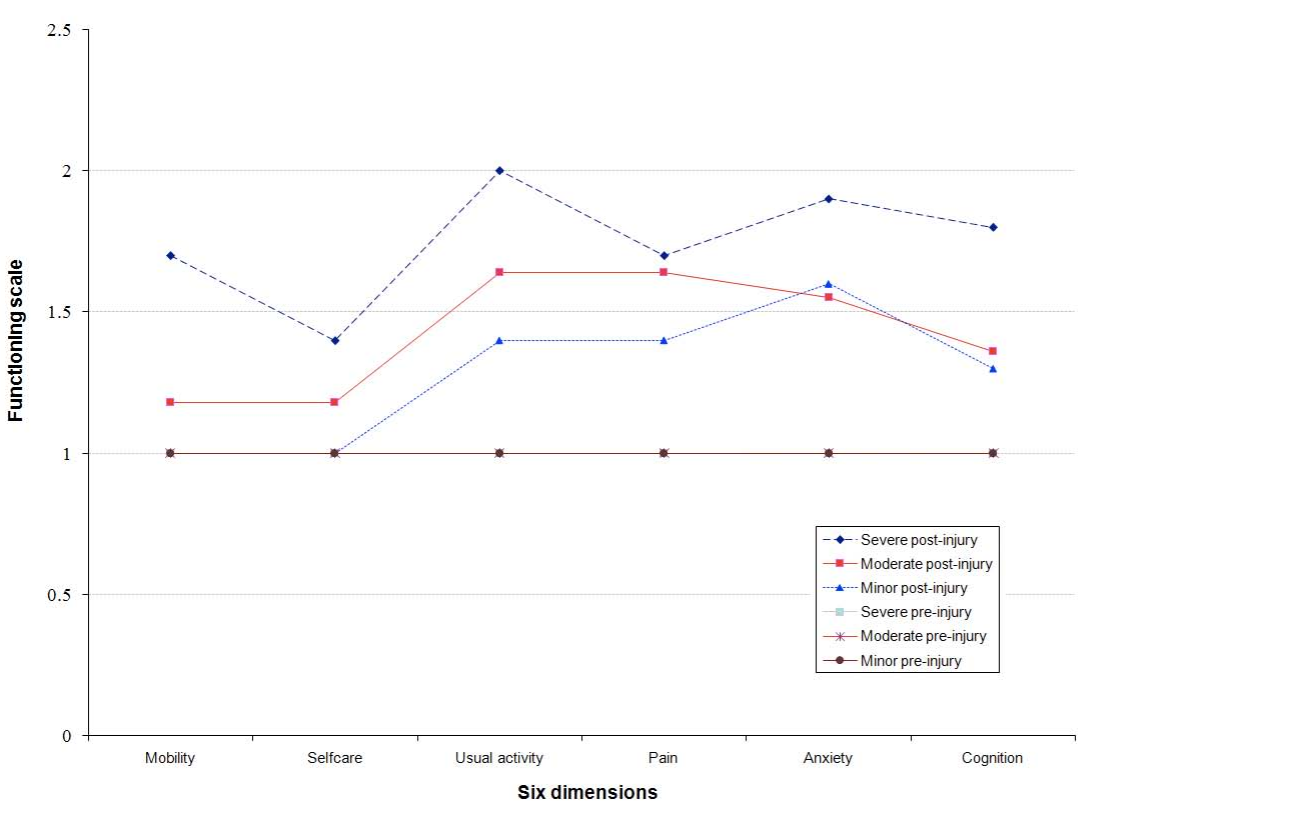
Change in ability to function in the six health dimensions.

Changes in quality of life were measured using the EQ6D instrument, administered to patients (or if unable to respond, to caregivers) in a questionnaire format. Disability again correlated with the severity of injury at admission. Patients with severe TBI were most compromised in their usual activities, with higher levels of anxiety and problems of cognition and mobility. All members of the moderate group faced disruption in their usual activities; with increased pain, anxiety and affected cognition. While none of the minor TBI patients faced difficulty in mobility and self-care, anxiety and pain were persisting problems, with continuing compromise of usual activities and cognition (Figure 1).

**Figure 2:**
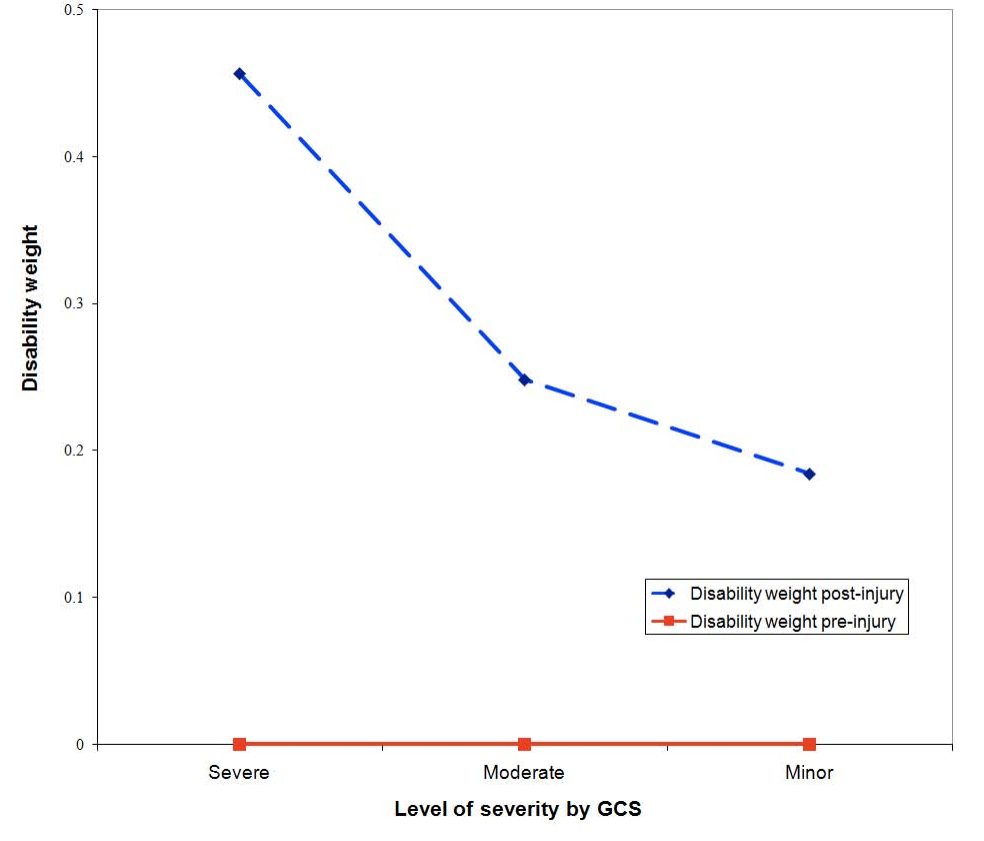
Predicted average disability weight under DALY form.

The average disability weights for TBI patients were assessed pre- and post-injury at the time of interview. While all patients shared the same disability weight of zero pre-injury, the disparity post injury reflected the level of severity (Figure 2). In term of intangible costs, the health related quality of life of the patients in the first year post-injury was reduced, resulting in an average year of life lost due to disability of 0.46 for severe, 0.25 for moderate and 0.15 year for minor TBI.

### Impact of TBI on family economic status

Eighty four percent of households in the sample faced treatment costs that accounted for more than 40% of the household capacity to pay for health care. The capacity to pay is determined by the remaining income of household after expenditure for basic subsistence needs. For this study, household health care expenditure that accounted for more than 40% of the household capacity to pay was taken to be catastrophic [3]. Only 12% of the households could afford to pay the cost associated with the treatment of TBI from household savings. The remaining households had to mobilize money for this payment from two or three sources, such as borrowing from relatives, using accumulated savings and/or selling assets, and resulting in financial stress at least in the medium term. Together with savings, support from relatives seemed to be the principle resource protecting households from catastrophic health expenditure.

## Discussion

This study is the first estimate of the costs of non-fatal TBI in motorcycle users not wearing helmets in Vietnam, in their first year post-injury. Given the limited coverage of health and social insurance in Vietnam, the study focused on out of pocket expenses and foregone earnings for both patients and their families. The cut-off after one year is a limitation of this study and contributes to an underestimation of the true total cost over a lifetime.

This study shows a large variance in the costs across individuals in the same level of severity, as seen in previous studies internationally and nationally [13-16], but confirms the significant level of financial burden that TBI imposes on families. It clearly demonstrates the direct correlation between level of severity of injury at admission and subsequent component costs, and the risk of catastrophic health expenditure for affected families.

As a pilot study using selected cost analysis methods, the study suggests that the use of per capita income to value the loss of productivity of TBI in Vietnam may underestimate indirect costs compared to estimates based on the individual's actual income. This reflects the reality that the majority of victims of motorcycle injuries are males within the economically productive age-group, and likely to be principal income earners for their households. As a result, their average income tends to be higher than the national income per capita. Since the costs of TBI in this study are confined to non-fatal TBI without complex complications, they must be considered as conservative estimates. Strategies such as withdrawing children from school to care for the injured, or to work in order to compensate for lost income have far reaching social consequences. The absence of accessible and affordable long-term rehabilitation is another concern concealed in these conservative estimates.

## Conclusion

This study has shown that all three components costs of TBI were high; the direct cost accounted for the largest proportion, with costs rising with the severity of TBI. The results suggest that the burden of TBI can be catastrophic for families because of high direct costs, significant time off work for patients and caregivers, and impact on health-related quality of life. Further research is warranted to explore the actual social and economic benefits of mandatory helmet use.

International experience shows that relatively affordable interventions such the implementation of mandatory helmet wearing for motorcycle riders result in the reduction of tangible and intangible costs to individuals, families and society [1]. Early unpublished data suggests that this is occurring in Vietnam. With the December 2007 introduction of mandatory helmet use, further research is now required to calculate the benefits of motorcycle helmet use in Vietnam together with research exploring compliance, quality standards and the development appropriate helmets for children. Such research will require larger sample sizes at each level of severity of TBI, covering different provinces and cities, targeting both use and non-use of helmets, and comparing different cost analysis methods. This research, however, already demonstrates a level of cost to individuals and households that is in many cases catastrophic, but which can be reduced through recognized policy interventions.

## Abbreviations

DALY: Disability Adjusted Life Years; DW: Disability weight; EQ6D: The European Quality of Life Instrument – 6 Dimensions; GCS: Glasgow Coma Scale; TBI: Traumatic Brain Injury; VND: Vietnamese Dong (currency; USD = 15,850 VND, July 2005); WHO: World Health Organization; YLD: Years lost due to disability.

## Competing interests

The authors declare that they have no competing interests.

## Authors' contributions

HTMH developed the literature review, clarified the research objective, developed instruments, interviewed the subjects, analysed the data, and completed the first draft.  TLP developed the literature review, clarified the research objective, developed instruments, interviewed the subjects, analysed the data and assisted with the first draft.  TTNV developed the literature review, clarified the research objective, developed instruments, interviewed the subjects, analysed the data and assisted with the first draft.  PKN negotiated local permission for the research, assisted in data analysis, and reviewed the draft. CMD and PSH conceived the study, assisted in the study design, instruments development and data analysis, reviewed and edited the draft.  All authors read and approved the final manuscript.
